# A multi-source molecular network representation model for protein–protein interactions prediction

**DOI:** 10.1038/s41598-024-56286-w

**Published:** 2024-03-14

**Authors:** Hai-Tao Zou, Bo-Ya Ji, Xiao-Lan Xie

**Affiliations:** 1 College of Information Science and Engineering, Guilin University of Technology, Guilin, 541000 China; 2 College of Computer Science and Electronic Engineering, Hunan University, Changsha, 410000 China

**Keywords:** Protein–protein interactions, Multi-source molecular network, Graph representation learning, Random forest, Bioinformatics, Biological models

## Abstract

The prediction of potential protein–protein interactions (PPIs) is a critical step in decoding diseases and understanding cellular mechanisms. Traditional biological experiments have identified plenty of potential PPIs in recent years, but this problem is still far from being solved. Hence, there is urgent to develop computational models with good performance and high efficiency to predict potential PPIs. In this study, we propose a multi-source molecular network representation learning model (called MultiPPIs) to predict potential protein–protein interactions. Specifically, we first extract the protein sequence features according to the physicochemical properties of amino acids by utilizing the auto covariance method. Second, a multi-source association network is constructed by integrating the known associations among miRNAs, proteins, lncRNAs, drugs, and diseases. The graph representation learning method, DeepWalk, is adopted to extract the multisource association information of proteins with other biomolecules. In this way, the known protein–protein interaction pairs can be represented as a concatenation of the protein sequence and the multi-source association features of proteins. Finally, the Random Forest classifier and corresponding optimal parameters are used for training and prediction. In the results, MultiPPIs obtains an average 86.03% prediction accuracy with 82.69% sensitivity at the AUC of 93.03% under five-fold cross-validation. The experimental results indicate that MultiPPIs has a good prediction performance and provides valuable insights into the field of potential protein–protein interactions prediction. MultiPPIs is free available at https://github.com/jiboyalab/multiPPIs.

## Introduction

Protein–protein interactions (PPIs) play an essential role in biological processes, such as cell metabolism, immune response^1^, and signal transduction^2^. Therefore, it is essential to develop effective strategies for correctly identifying potential PPIs to understand better protein functions and model complex protein structures. In recent years, some small-scale experimental methods (such as chromatography and biochemical assays) are always utilized to predict the potential PPIs. However, these methods are often inefficient, high time-consuming, and not suitable for large-scale prediction^3^. Hence, several high-throughput experimental methods have also been invented for identifying potential protein–protein interactions, including immune precipitation, yeast two-hybrid screens (Y2H)^4^, crystallography, and protein chips^5^. These methods have generated copious known protein–protein interaction pairs, which is of great importance for analyzing potential PPIs. Nevertheless, these high-throughput technologies still have obvious drawbacks, such as a high false-positive rate, small coverage, and time-intensive^6,7^. Accordingly, due to these limitations of traditional experimental methods, there is an urgent need to develop effective and accurate computational models to identify potential PPIs. In recent years, more and more computational methods^8–12^ have been developed as an aid to biological experiment methods with the aim of solving their high false-positive, small converge and time-intensive problems. More specifically, computational methods employ sophisticated algorithms and statistical models to analyze biological data, helping to minimize false-positive results^8–12^. They take advantage of the availability of vast amounts of biological data generated through high-throughput techniques. By analyzing large-scale datasets, these methods can identify patterns, trends, and associations that may be undetectable with traditional experimental approaches. Furthermore, biological experiments can be time-consuming and costly, requiring extensive sample preparation, data collection, and analysis. Computational methods provide a more efficient and cost-effective alternative. Once the necessary algorithms and models are developed, computational analyses can be performed relatively quickly on powerful computer systems. This saves time and resources, allowing researchers to explore a broader range of hypotheses or conduct large-scale investigations more feasibly.

Recently, several computational methods for potential protein–protein interaction prediction have been proposed. Of these, some methods take advantage of 3D structure^13^, gene ontology and annotations^14^, gene fusion, and co-expression^15–18^ technologies. However, these technologies usually require prior knowledge of the collected proteins, which dramatically limits their accuracy and reliability. For example, the 3D structure of many proteins is difficult to obtain, and the gene ontology annotation of proteins is incomplete^19–23^. In contrast, abundant sequence data of proteins from multiple sources is relatively easy to obtain. Thence, several computational methods based on sequence features of proteins have been developed to predict potential PPIs. For example, Shen et al*.*^24^ developed a novel model for protein–protein interaction prediction only utilizing protein sequence information. In their work, protein sequence information was first extracted based on amino acids' triad characteristics. Then the model was constructed by using support vector machines (SVM) combined with a kernel function. This experiment fully proves that the computational methods only using protein sequence features also have a good prediction ability of protein–protein interactions. Guo et al*.*^25^ constructed a new protein sequence feature representation method to predict potential PPIs. Specifically, they selected the auto covariance (AC) method to extract the characteristics of protein sequences based on seven physicochemical properties of amino acids. This method thoroughly considered the interactions between amino acids at different distances in the protein sequence and ultimately performed better than other sequence-based methods. Furthermore, their study demonstrated that extracting protein sequence features by the auto-covariance (AC) method is feasible and effective for potential protein–protein interactions prediction.

In addition, machine learning algorithms have also attracted the attention of many researchers in the field of potential protein–protein interactions prediction. For example, Wang et al*.*^26^ developed a feature-weighted Rotation Forest model for protein–protein interaction prediction by eliminating useless information to use the valuable features fully. In the results, their proposed method achieved excellent prediction performance under the cross-validation experiment. You et al*.*^27^ presented a new method to transform the protein sequence features into matrix representation and then utilized the support vector machine (SVM) for training and prediction. Their model finally achieved excellent prediction results in the yeast PPIs datasets. Finally, You et al*.*^28^ developed an ensemble weighted sparse representation model classifier and replaced the matrix representation with the integrated protein sequence-function to predict potential protein–protein interactions. Compared with many previous advanced methods, this model has better performance.

**Figure 1:**
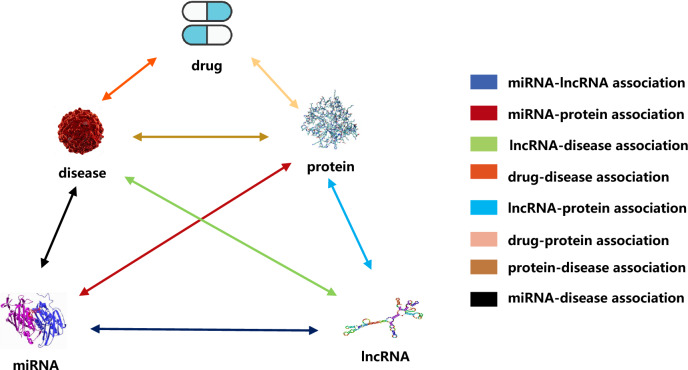
The multi-source molecular network.

**Figure 2:**
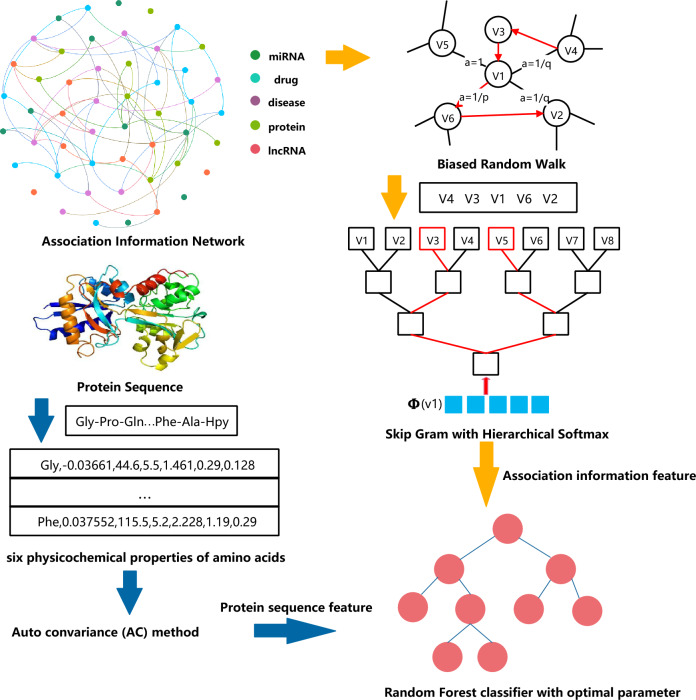
The flowchart of our proposed model.

Human cells are part of a complex biomolecular network, involving interactions and associations among various biomolecules, such as proteins, miRNAs, and diseases. Proteins often interact with each other based on their shared relationships with other biomolecules. Leveraging this associated information can help predict potential protein–protein interactions (PPIs). In this study, we introduce a new computational model (called MultiPPIs) to predict PPIs. This model combines protein sequence physicochemical features with multi-source biomolecular association data (including drugs, miRNAs, lncRNAs, and diseases). First, we use the auto-covariance method to extract features from protein sequences based on amino acids' physicochemical properties. Second, we create a network that integrates known associations among various biomolecules, as depicted in Fig. 1. Using DeepWalk^29^, a graph representation method, we extract association information from this network. We then utilize 19,237 known PPI pairs from the STRING database (2017)^30^ as our positive dataset. A matching number of random non-interacting pairs form the negative dataset. These datasets are combined to create our final training set. The prediction model is constructed using a Random Forest (RF) classifier, optimized for best performance. The process flow of MultiPPIs is outlined in Fig. 2. In our study, the proposed model, under fivefold cross-validation, achieves an average accuracy of 0.8603 and an AUC of 0.9304. These results are better than many current computational methods. We also compared two feature combination strategies. Our method is more effective than using only protein sequence information by combining multiple types of data. Additionally, we test four popular classifiers and find the Random Forest classifier to be the most suitable for our model, offering superior prediction performance. These experiments demonstrate that our model is an efficient tool for predicting potential protein–protein interactions. Compared with previous computational methods^8–12^, our method mainly has the following specific advantages: (1) Considering the holistic nature of biomolecular networks, our method collects a large amount of association data to construct a multi-source molecular network, and extracts the higher-order network features of proteins based on the graph representation learning method to improve the accuracy of the prediction of PPIs. (2) Our method fully takes advantage of the local property of residues in protein sequences and describes the level of correlation between two protein sequences based on their specific physical and chemical properties. This not only improves the prediction performance of our method, but also solves the cold-start problem often encountered by graph neural network-based methods. (3) By conducting extensive experiments, including comparison of feature combinations, comparison of classification models, optimization and adjustment of model parameters, and comparison with previous experimental methods, our method has been confirmed to have excellent performance in predicting PPIs and is better than most previous computational methods.

## Results and discussion

### The five-fold cross-validation performance of our proposed model

Cross-validation is a standard method used in machine learning to construct and validate model parameters. In this work, fivefold cross-validation was adopted to evaluate the performance of our model. First, we equally divided the sample data into five parts. Second, we sequentially selected four parts as the training set and the remaining 1 part as the test set. The experiment repeated 5 times. Finally, six standard parameters were used as evaluation indicators for our experiments, including specificity (Spec.), Matthews's correlation coefficient (MCC), precision (Prec.), sensitivity (Sen.), accuracy (Acc.), and the areas under the ROC curve (AUC). Table 1 lists the detailed results of each validation. The last line shows the average value and the standard deviation of the results across five runs of the classifier. These experimental results demonstrated that our model could achieve good results and stability in the protein–protein interaction prediction.

**Table 1:** The fivefold cross-validation results of our proposed model.

Folder	Spec. (%)	MCC (%)	Prec. (%)	Sen. (%)	ACC. (%)	AUC (%)
0	89.97	73.62	89.27	83.50	86.73	93.55
1	89.60	73.21	88.93	83.47	86.54	93.37
2	90.07	71.59	89.11	81.24	85.65	93.15
3	88.96	72.09	88.26	83.00	85.98	92.81
4	88.27	70.66	87.52	82.26	85.27	92.30
Average	89.37 ± 0.76	72.23 ± 1.20	88.62 ± 0.72	82.69 ± 0.95	86.03 ± 0.61	93.03 ± 0.49

**Figure 3:**
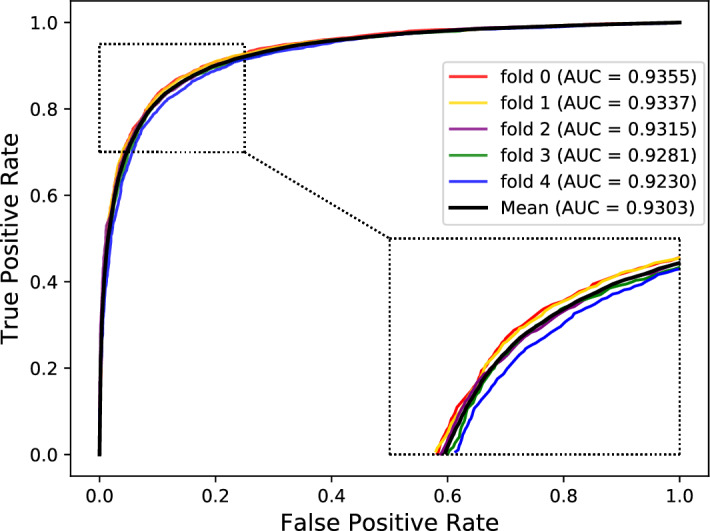
The ROC curves and AUC values of our model under fivefold cross-validation.

**Figure 4:**
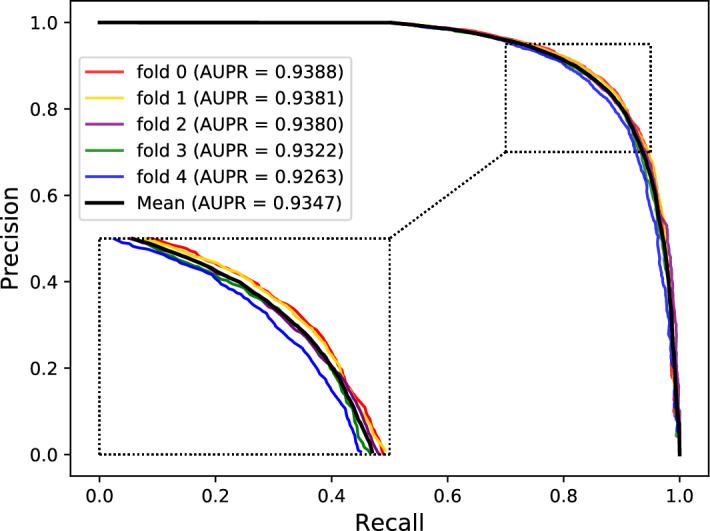
The PR curves and AUPR values of our model under fivefold cross-validation.

The Receiver Operating Characteristic (ROC) curve is an essential and common statistical analysis tool widely used to judge the quality of classification and prediction results in medical research and machine learning. It first sorts the samples according to the prediction results of the classifier and then predicts the samples as positive samples one by one in this order. This way calculates two important values (True Positive Rate, False Positive Rate) each time and plots them as the horizontal and vertical coordinates, respectively. Besides, the AUC is defined as the areas under the ROC curve, and its value range is generally between 0.5 and 1. Generally, the ROC curve cannot indicate which classifier has better performance, so the AUC value is selected as the evaluation index. The classifier with a larger AUC has better performance. The Precision-Recall (PR) curve is another tool to evaluate the performance of a classifier. For the category imbalance problem, the PR curve is widely considered superior to the ROC curve. Similarly, the AUPR is defined as the areas under the PR curve. Figures 3 and 4 respectively show our method's ROC and PR curves under fivefold cross-validation. These results once again demonstrated our model's good effect and stability in predicting potential protein–protein interactions.

### Compare the effect of our feature combination strategy

**Figure 5:**
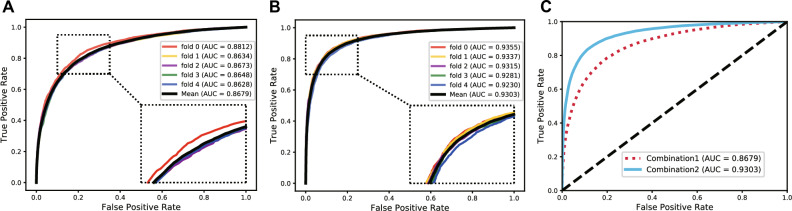
The ROC curves and AUC values of two different feature combination strategies. (**A**) the ROC curves and AUC values of the only protein sequence features. (**B**) The ROC curves and AUC values of the combination of protein sequence features and the multi-source associated information of proteins. (**C**) Comparison of the ROC curves and AUC values of two different feature combination strategies.

**Figure 6:**
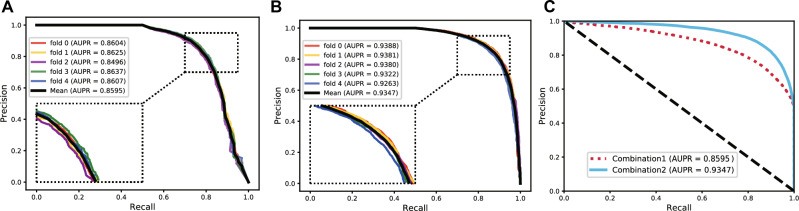
The PR curves and AUPR values of two different feature combination strategies. (**A**) The PR curves and AUPR values of the only protein sequence features. (**B**) The PR curves and AUPR values of the combination of protein sequence features and the multi-source associated information of proteins. (**C**) Comparison of the PR curves and AUPR values of two different feature combination strategies.

To further compare the effect of our feature combination strategy, a different feature combination was utilized to represent protein nodes. More specifically, we used the only protein sequence features (combination 1) and the combination of the protein sequence features and the multi-source associated information of proteins used by MultiPPIs (combination 2) to represent proteins before carrying out the fivefold cross-validation experiment. One important thing that must be mentioned is that the experimental environment of the two different combinations is the same to ensure the fairness of comparison. Table 2 lists the results of the experiment results of combination 1 under the fivefold cross-validation experiment. The experiment results of combination 1 is shown in Table 1. Figures 5 and 6, respectively, show the comparative experiment's ROC curves and PR curves. As the results show, our feature combination strategy performs better than most computational methods that only use protein sequence features. This once again proves that the multi-source association information with other biomolecules of proteins is helpful for protein–protein interaction prediction.

**Table 2:** The results of different feature combinations under fivefold cross-validation.

Feature	Folder	Spec.(%)	MCC(%)	Prec.(%)	Sen.(%)	ACC.(%)	AUC(%)
Combination 1	0	80.64	61.02	80.59	80.38	80.51	88.12
	1	78.64	57.56	78.70	78.92	78.78	86.34
	2	79.44	57.10	79.07	77.65	78.55	86.73
	3	78.92	57.93	78.94	79.00	78.96	86.48
	4	79.79	57.98	79.46	78.18	78.99	86.28
	Average	79.49 ± 0.78	58.32 ± 1.55	79.35 ± 0.74	78.83 ± 1.03	79.16 ± 0.78	86.79 ± 0.76

### Compare the effect of different classifiers

**Figure 7:**
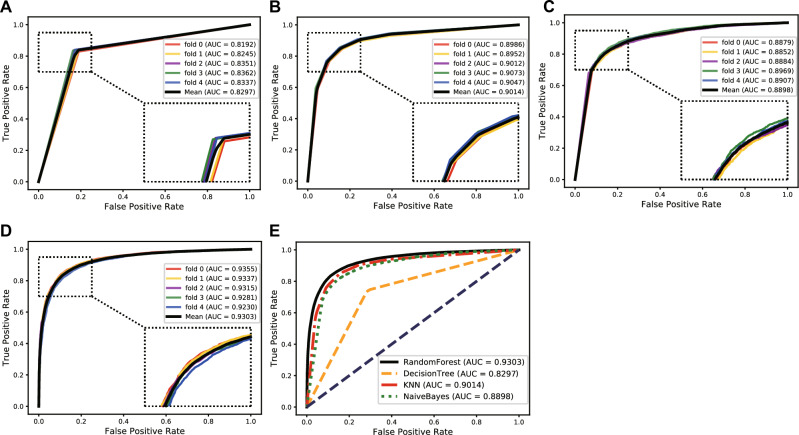
The ROC curves and AUC values of different classifiers. (**A**) The ROC curves and AUC values of the Decision Tree classifier. (**B**) The ROC curves and AUC values of the KNN classifier. (**C**) The ROC curves and AUC values of the Naive Bayes classifier. (**D**) The ROC curves and AUC values of the random forest classifier. (**E**) Comparison of the ROC curves and AUC values of different classifiers.

**Figure 8:**
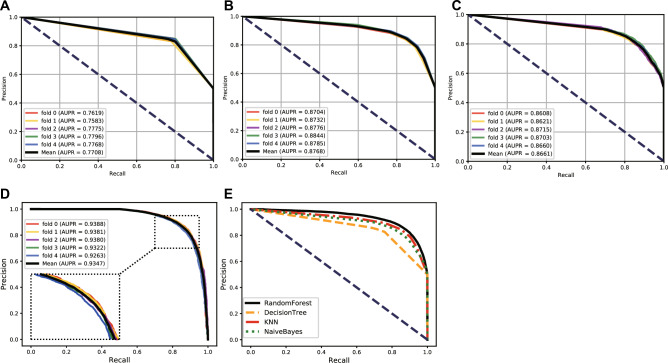
The PR curves and AUPR values of different classifiers. (**A**) The PR curves and AUPR values of the decision tree classifier. (**B**) The PR curves and AUPR values of the KNN classifier. (**C**) The PR curves and AUPR values of the Naive Bayes classifier. (**D**) The PR curves and AUPR values of the Random Forest classifier. (**E**) Comparison of the PR curves and AUPR values of different classifiers.

To choose the most suitable classifier for our model, we conducted a comparison experiment with the four most commonly used classifiers, including Decision Tree, Naive Bayes, KNN, and Random Forest. We used these four classifiers with default training parameters to train and predict the protein–protein interactions and kept other experimental conditions consistent. Finally, the Random Forest classifier performed better by observing the prediction results. Table 3 lists the average parameter values of different classifiers under fivefold cross-validation. Figures 7 and 8, respectively, show the ROC and PR curves of the comparative experiment. The comparison experiment results proved that the Random Forest is more suitable for our model than other classifiers, especially in terms of the AUC and accuracy, which can represent the ability of a model.

**Table 3:** The average parameter values of different classifiers under fivefold cross-validation.

Classifier	Spec. (%)	MCC. (%)	Prec. (%)	Sen. (%)	ACC. (%)	AUC. (%)
DecisionTree	77.47 ± 0.82	60.76 ± 1.30	78.25 ± 0.69	80.27 ± 0.63	78.87 ± 0.65	82.97 ± 0.65
KNN	84.39 ± 0.71	69.42 ± 1.07	84.49 ± 0.63	85.03 ± 0.59	84.71 ± 0.53	90.14 ± 0.48
Naive Bayes	82.73 ± 0.66	66.08 ± 1.05	82.84 ± 0.53	83.34 ± 0.95	83.04 ± 0.53	88.98 ± 0.44
RandomForest	89.37 ± 0.76	72.23 ± 1.20	88.62 ± 0.72	82.69 ± 0.95	86.03 ± 0.61	93.03 ± 0.49

### Compare the effect of random forest classifier parameter

Random Forest (RF) is a flexible and efficient supervised learning algorithm Breiman proposed in 2001. This algorithm has achieved good results in solving problems in many fields. It has the characteristics of preventing overfitting, strong model stability, and easy to deal with nonlinear regression problems. It is also a particular bootstrap aggregating (bagging) method which uses the decision tree as the training model. It first uses the bootstrap method to generate training sets and then constructs a decision tree for each training set. Finally, all these decision trees are combined to form the classifier to improve the overall effect. Additionally, when segmenting node features, the Random Forest method does not select all features that can maximize the index (such as information gain). Instead, it randomly extracts a subset of features and then finds the optimal solution within this subset. For the Random Forest model parameters, we need to set the regression tree number N. In detail, and we started to train the model at an interval of 20 from *N* = 180 and observed the relationship between the number of *N* and the final prediction accuracy. We terminated the model training if the prediction accuracy decreased with the increase of *N*. Table 4 lists the accuracy results of the Random Forest classifier with different *N* parameters under fivefold cross-validation. As a result, we can see that the Random Forest classifier has the best performance when the number of regression trees is 300.

**Table 4:** The accuracy results of the Random Forest classifier with different *N* parameters.

Fold	0	1	2	3	4	Average
$$N$$						
180	0.8647	0.8654	0.8565	0.8577	0.8505	0.8590 ± 0.62
200	0.8647	0.8627	0.8559	0.8578	0.8476	0.8577 ± 0.67
220	0.8646	0.8643	0.8572	0.8577	0.8498	0.8587 ± 0.61
240	0.8633	0.8677	0.8590	0.8599	0.8477	0.8595 ± 0.74
260	0.8671	0.8649	0.8588	0.8598	0.8469	0.8595 ± 0.78
280	0.8663	0.8664	0.8572	0.8603	0.8490	0.8598 ± 0.72
300	0.8673	0.8654	0.8565	0.8598	0.8527	0.8603 ± 0.61
320	0.8645	0.8628	0.8589	0.8604	0.8505	0.8594 ± 0.54
340	0.8658	0.8649	0.8562	0.8578	0.8495	0.8588 ± 0.67

### Performance comparison with the state-of-the-art methods

To further evaluate the effectiveness of MultiPPIs, we conduct a detailed comparative analysis between it and several existing protein–protein interaction prediction methods, including LR_PPI^31^, DPPI^32^, WSRC_GE^33^, LPPI^34^ and PIPR^35^. Our evaluation framework encompasses five distinct performance metrics, as detailed in Table 5. These metrics include specificity (Spec.), Matthews’s correlation coefficient (MCC), precision (Prec.), sensitivity (Sen.), accuracy (Acc.), and the areas under the ROC curve (AUC), providing a comprehensive view of each method's predictive capabilities. Our findings reveal a significant enhancement in performance with MultiPPIs. This substantial leap in accuracy underscores the effectiveness of MultiPPIs in identifying protein–protein interactions, marking a notable advancement in the field.

**Table 5:** Performance comparison of MultiPPIs with the state-of-the-art methods.

Methods	AUPR.(%)	Prec.(%)	Sen.(%)	ACC.(%)	AUC.(%)
LR_PPI^31^	84.11 ± 0.58	73.29 ± 0.92	75.51 ± 0.90	77.17 ± 0.66	84.82 ± 0.60
DPPI^32^	89.03 ± 0.78	76.77 ± 0.90	76.23 ± 0.99	80.07 ± 0.87	87.26 ± 0.76
WSRC_GE^33^	89.75 ± 0.86	79.87 ± 1.23	76.23 ± 0.97	82.25 ± 1.05	90.22 ± 0.89
LPPI^34^	80.22 ± 1.54	72.32 ± 1.03	82.75 ± 1.24	80.62 ± 1.16	84.24 ± 1.73
PIPR^35^	82.46 ± 0.96	74.56 ± 0.98	76.78 ± 1.00	75.36 ± 0.90	83.31 ± 0.94
**MultiPPIs**	93.08 ± 0.45	88.62 ± 0.72	82.69 ± 0.95	86.03 ± 0.61	93.03 ± 0.49

## Materials and methods

### Protein sequence features based on the physicochemical properties of amino acids

In this study, we downloaded the sequence information of proteins from the STRING: in 2017^30^ database. Proteins are biopolymers composed of up to 20 different amino acids as basic units. The sequence of amino acid residues in the peptide chain is called the primary structure of proteins. Consequently, we selected the six physicochemical properties of amino acids to represent the protein sequence features in this work, including polarity (P1), hydrophobicity (H), net charge index of side chains (NCISC), volumes of side chains of amino acids (VSC), solvent-accessible surface area (SASA) and polarizability (P2). The original physicochemical values of these 20 amino acids are listed in Table 6.

**Table 6:** The original physicochemical values of 20 amino acids.

Amino acids	NCISC	VSC	P1	SASA	H	P2
Cys	− 0.03661	44.6	5.5	1.461	0.29	0.128
Asp	− 0.02382	40	13	1.587	− 0.9	0.105
Glu	0.006802	62	12.3	1.862	− 0.74	0.151
Ile	0.021631	93.5	5.2	1.81	1.38	0.186
Gly	0.179052	0	9	0.881	0.48	0
Leu	0.051672	93.5	4.9	1.931	1.06	0.186
Val	0.057004	71.5	5.9	1.645	1.08	0.14
Met	0.002683	94.1	5.7	2.034	0.64	0.221
Trp	0.037977	145.5	5.4	2.663	0.81	0.409
Asn	0.005392	58.7	11.6	1.655	− 0.78	0.134
His	− 0.01069	79	10.4	2.025	− 0.4	0.23
Gln	0.049211	80.7	10.5	1.932	− 0.85	0.18
Ala	0.007187	27.5	8.1	1.181	0.62	0.046
Arg	0.043587	105	10.5	2.56	− 2.53	0.291
Tyr	0.023599	117.3	6.2	2.368	0.26	0.298
Pro	0.239531	41.9	8	1.468	0.12	0.131
Lys	0.017708	100	11.3	2.258	− 1.5	0.219
Ser	0.004627	29.3	9.2	1.298	− 0.18	0.062
Thr	0.003352	51.3	8.6	1.525	− 0.05	0.108
Phe	0.037552	115.5	5.2	2.228	1.19	0.29

### Performance evaluation criteria for our experiments

In order to verify the quality of our proposed method, six standard parameters were calculated as evaluation indicators for our experiments, including specificity (Spec.), Matthews's correlation coefficient (MCC), precision (Prec.), sensitivity (Sen.), accuracy (Acc.), and the areas under the ROC curve (AUC). The description of all computational formulas is as follows:

1 $$Spec =\frac{TN}{FP+TN}$$

2 $$MCC=\frac{TP\times TN-FP\times FN}{\surd (TP+FN)\times (TN+FP)\times (TP+FP)\times (TN+FN)}$$

3 $$Prec =\frac{TP}{FP+TP}$$

4 $$Sen =\frac{TP}{TP+FN}$$

5 $$Acc =\frac{TP+TN}{TP+FP+TN+FN}$$

where TN, FN, TP, and FP represent the total number of true negative, false negative, true positive, and false positive. Furthermore, the AUC (the area under the ROC curve) was also implemented to evaluate the performance of our model.

### Auto covariance (AC) method

The extraction of protein sequence features using the auto covariance (AC) method was completely proposed by Guo et al.^36^. This method fully takes advantage of the local property of residues in protein sequences and describes the level of correlation between two protein sequences based on their specific physical and chemical properties^37–39^. First, we normalized the original physicochemical values of 20 amino acids to unit standard deviations (SD) and zero means according to Eq. (1):

6 $${{P}_{ij}}^{\mathrm{^{\prime}}}=\frac{{P}_{ij}-\overline{{P }_{j}}}{{S}_{j}}, (i=\mathrm{1,2},\dots ,6;j=\mathrm{1,2},\dots 20)$$

where ${P}_{ij}$ is the ${j}_{th}$ descriptor value for ${i}_{th}$ amino acid, $\overline{{P }_{j}}$ is the mean of ${j}_{th}$ descriptor over the 20 amino acids and ${S}_{j}$ is the corresponding standard deviations, given by:

7 $$\overline{{P }_{j}}=\frac{{\sum }_{i=1}^{20}{P}_{ij}}{20}$$

8 $${S}_{j}= \sqrt{\frac{{\sum }_{i=1}^{20}{({P}_{ij}-\overline{{P }_{j}})}^{2}}{20}}$$

In this way, each amino acid in a protein sequence is converted to the corresponding standardized physicochemical value. Then, the AC method is used to encode the protein sequence into a feature vector:

9 $${\text{AC}}=\frac{1}{N-d}{\sum }_{j=1}^{N-d}({X}_{i,j}-\frac{1}{n}\sum_{i=1}^{n}{X}_{i,j})({X}_{i+d,j}-\frac{1}{n}\sum_{i=1}^{n}{X}_{i,j})$$

where ${X}_{i,j}$ is the ${j}_{th}$ descriptor value of the ${i}_{th}$ amino acid, *N* is the length of the protein sequence, *d* is the width of the sliding window. In this article, the parameters *d* and *j* are respectively set to 30 and 6. On this basis, a protein sequence is finally encoded as a 30*6 = 180-dimensional feature vector.

### The multi-source molecular network construction

In order to utilize the associated information of proteins with other biomolecules, we systematically and comprehensively constructed the association information network by integrating the known associations among proteins, diseases, miRNAs, drugs, and lncRNAs, which were downloaded from multiple databases. The source and version of the raw data are shown in Table 7 below. In addition, we have done some operations with the raw data, such as removing some irrelevant items and unifying the identifiers. Besides, we also counted the number of nodes contained in the original association data, as shown in Table 8.

**Table 7:** The data information in the multi-source molecular network.

Association information	Database	Amount
miRNA-disease	HMDD v3.0^40^	16,427
drug-protein	DrugBank v5.0^41^	11,107
miRNA-lncRNA	lncRNASNP2^42^	8374
lncRNA-disease	lncRNASNP2^42^, LncRNADisease^43^	1264
drug-disease	CTD: updata 2019^44^	18,416
protein-disease	DisGeNET^45^	25,087
miRNA-protein	miRTarBase: updata 2018^46^	4944
lncRNA-protein	LncRNA2Target v2.0^47^	690
Total	N/A	86,309

**Table 8:** The node information in the multi-source molecular network.

Node	Amount
LncRNA	769
Protein	1649
MiRNA	1023
Drug	1025
Disease	2062
Total	6528

### DeepWalk algorithms

In order to extract the associated information feature of proteins from the association information network we constructed, the graph embedding algorithms: DeepWalk^29^ was adopted in our work. The input of the DeepWalk method is a graph or network, and then the social representation of vertices in the network was learned through the truncated random walk and the SkipGram model. Finally, it outputs the potential relationship of vertices in the network. The basic idea of this algorithm is first to obtain the node sequence as a sentence through the random walk, and then to obtain the local information of the network from the truncated random walk sequence by maximizing the co-occurrence probability of vertex ${v}_{j}$ within a window size *w* to learn the potential representation of the node based on the local information, which is calculated as follows:

10 $$\Pr \left( {\left\{ {v_{j - w} , \ldots ,v_{j + w} } \right\}s\backslash v_{j} |\Phi \left( {v_{j} } \right)} \right) = \prod\nolimits_{i = j - w,i \ne j}^{j + w} {\Pr \left( {v_{i} |\Phi \left( {v_{j} } \right)} \right)}$$

11 $${\text{Pr}}({v}_{i}|\Phi \left({v}_{j}\right)=\prod_{k=1}^{\left[{\text{log}}\left|V\right|\right]}1/(1+{e}^{-\Phi \left({v}_{j}\right)\cdot \varphi \left({b}_{k}\right)})$$

where $\Phi ({v}_{j})$ indicates that vertex ${v}_{j}$ is mapped to its representation space, $\varphi ({b}_{k})$ means the parent node of the tree node ${b}_{k}$. More specifically, the entire DeepWalk method is mainly composed of two algorithms. Algorithm 1 of the DeepWalk model mainly includes 4 steps: (1) Generate γ random walks for each node in the input network structure. (2) Uniformly samples a point in the network as the root node in each random walk process. (3) Uniformly select the neighbor node as the next node from the current node. (4) Repeat the above steps until the walking path reaches the specified length. Algorithm 2 of the DeepWalk model is to perform the SkipGram model for training the sequence data to get the network feature vector of each node. The SkipGram model iters all possible matches within a window for the random walk sequence. It utilizes nodes to assume the context and discovers the representation of the vector by achieving the maximum co-occurrence probability of words in a window while neglecting the order in which the nodes occur in the sentence. According to the independent presumption, the probability of co-occurrence can be transferred into the conditional probability product. The detailed process of the algorithm is respectively shown in Tables 9 and 10. In this way, the associated information with other biomolecules of proteins in the association information network is converted to the feature vector, which can be used by the machine learning classifiers.

**Table 9:**
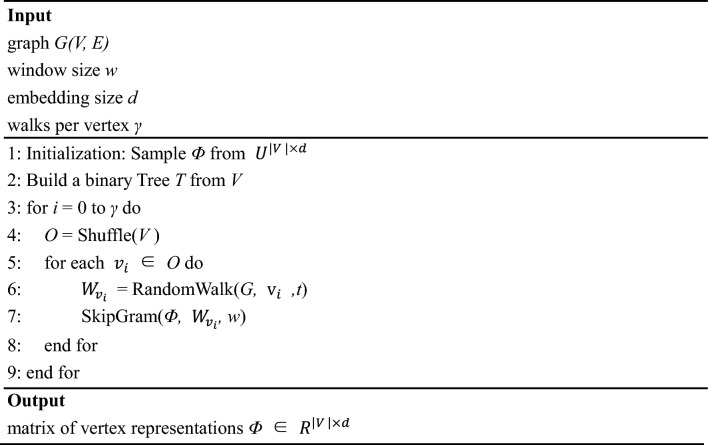
Algorithm 1 of the DeepWalk model.

**Table 10:**
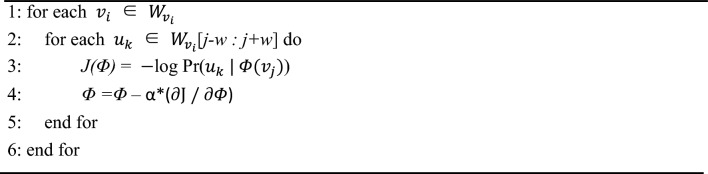
Algorithm 2 of the DeepWalk model

### The representation of protein nodes

In this study, the protein nodes were represented by the combination of the physicochemical features of protein sequences and multi-source association information with other biomolecules (drugs, miRNAs, lncRNAs, and diseases) of proteins in the association information network. The sequence feature of proteins was obtained by the auto-covariance (AC) method based on the six physicochemical properties of amino acids. Besides, the associated information with other nodes of proteins was obtained by the network representation method DeepWalk based on the association information network we constructed. Finally, we combined these two features to represent the protein–protein interaction pairs.

## Conclusion

The protein–protein interactions (PPIs) play a vital role in the cell biochemical reaction network and are significant for regulating cells and their signals. However, the traditional biological experiment methods have the limitations of a high time-consuming and long period, which is not suitable for large-scale protein–protein interactions prediction. In this study, we proposed a novel computational method to predict potential PPIs by combining the sequence feature and associated information with other molecules of proteins. For the sequence feature of proteins, we utilized the auto covariance (AC) method to extract it based on the six physicochemical properties of amino acids. For the association information feature with other molecules of proteins, we utilized the DeepWalk network representation method to extract it based on the association information network we constructed. In this way, the proteins were represented by combining these two features. Finally, the Random Forest classifier and its corresponding optimal parameters were selected for training and prediction. As a result, our proposed method achieved average accuracy and AUC of 86.03% and 93.03% under fivefold cross-validation, which is superior to many existing computational models. Besides, to evaluate the effect of our feature combination, we further compared the performance of only the protein sequence feature and the combination of protein sequence and association feature. Furthermore, to select the most suitable classifier for our model, we also compared the ability of the four most commonly used classifiers. While overcoming many challenges, our current method still has its limitations. In our work, we collected 8 associations between 5 biological molecules to construct a multi-source molecular network. All the proteins in our dataset are distributed on this network. Therefore, we are able to utilize the relationships between different molecules to extract the network features of protein nodes. Note that we have removed known protein–protein interactions during training to avoid causing label leakage. An independent test set, completely independent of the existing dataset, would result in the inability to use molecular network relationships. We designed our model to address this limitation by considering both the physicochemical properties of the protein sequence. For new proteins that cannot be added to the network, we use this feature for interaction prediction. Our data and code is open source, easily available at https://github.com/jiboyalab/multiPPIs.

## Data Availability

The data and source code are available in a public github repository: https://github.com/jiboyalab/multiPPIs
